# The Wages of Prosperity

**Published:** 1900-03-17

**Authors:** 


### THE WAGES OF PROSPERITY.

Dr. Dawson Burns has, with his usual exactitude, again set forth in the *Times* the national drink bill for the past year, and undoubtedly the steady growth in the figures in question is a matter of considerable national importance. It is easy to say that a nation which can spend £162,163,474 on alcoholic drinks in a single year, as we did last year, must be in a good financial position; and to deduce the cheering inference that we need not be troubled about wars and other difficulties, seeing that we have but to save this waste, and our balance will come right again. But shall we save it? The nation is but a vast congeries of individuals; and experience in regard to individuals teaches that when the pinch comes the use of alcoholic drinks, instead of being one of the first, is one of the very last indulgences to be renounced. It is in this that, so far as finance is concerned, the gravity of this enormous expenditure really lies. The habit of depending upon alcohol for a part of one's daily output of energy, and for a very considerable part of one's sense of well-being, is one which is by no means easily broken, and which, as a fact, is often not broken even when the strongest reasons exist for so doing, so that we can feel but little confidence in our drink bill as a reserve of national potential wealth. In the meantime we cannot but see how serious is likely to be the effect upon the national health and character of such a large and steadily increasing consumption of alcohol as is indicated by the figures given. When we are told that the estimated expenditure upon alcohol per family of five persons during the year 1899 was within a few pence of £20, and that, reducing the various drinks to their equivalents in alcohol, the average amount of this substance drunk in England came to an average of 2·51 gallons per head of the population, we see that we have reached a consumption which even those who have no belief in the physiological or moral advantages of teetotalism must admit to be excessive. Taking the figure arrived at by Parkes, Anstie, and others, as the amount of alcohol which may be safely taken per day, namely, between one and two ounces, we see how largely that has already been exceeded by alcohol drinkers in England. Two and a half gallons means 400 ounces in the year, or more than an ounce a day for every man, woman, and child in the country, so that if we deduct the children, and, as we hope we may do, a considerable number of the women, together with the growing number of abstainers, and then divide the total consumption among the remainder, we see that the existence of a very large amount of habitual excess is a matter of demonstration, and when it is further stated that this expenditure on alcoholic drinks is a growing one, and that the amount so spent in the United Kingdom was £6,169,455 more in 1899 than it was in the previous year, we have said enough to show that, if this is the way in which the wages of prosperity are being scattered, perhaps a turn of adversity might not be without its compensating advantages.

